# Pseudobulbar Affect Among Patients With Dementia

**DOI:** 10.7759/cureus.78116

**Published:** 2025-01-28

**Authors:** Lily Charron, Eduardo D Espiridion

**Affiliations:** 1 Psychiatry, Drexel University College of Medicine, West Reading, USA; 2 Psychiatry, Drexel University College of Medicine, Philadelphia, USA; 3 Psychiatry, Reading Hospital - Tower Health, West Reading, USA

**Keywords:** dementia, loss of memory, neurocognitive disorder, postconcussion syndrome, pseudobulbar affect

## Abstract

Objective: Dementia is a neurocognitive disorder characterized by memory loss and deficits in multiple cognitive domains, caused by damage to or loss of neurons in the brain, which impairs one’s abilities and capabilities for independent daily living. The etiologies of dementia are diverse, including vascular, infectious, traumatic, and neurodegenerative causes. The most common types of dementia include Alzheimer’s dementia, vascular dementia, Lewy body dementia, and frontotemporal dementia. Patients with dementia frequently develop cognitive, psycho-behavioral, emotional, and mood symptoms. One largely unstudied mood symptom seen in dementia patients is pseudobulbar affect (PBA). PBA is a state of emotional incontinence characterized by episodes of uncontrolled crying or laughter that are inconsistent with the social context or the patient’s emotional state. Although many neurological disorders may present with PBA, only a small fraction of the literature focuses on PBA in dementia patients. In the present study, we used the TriNetX database to identify a cohort of patients with both PBA and dementia. We describe this cohort to provide a foundation for further research on this patient population.

Results: Seventy-three percent (n=182) of the cohort had postconcussion syndrome, 18% (n=44) had overt dementia, 49% (n=121) had mild cognitive impairment, and 42% (n=105) had some form of amnesia. Many patients had comorbid psychiatric disorders, including anxiety and depressive disorders.

Conclusions: The multitude of comorbid mood disorders and symptoms can complicate the clinical management of dementia patients, adding to their distress and that of their caregivers. Understanding these symptoms is essential for providing an accurate diagnosis and effective management of PBA in dementia.

## Introduction

Dementia is a major neurocognitive disorder associated with significant memory loss and cognitive decline. Though commonly associated with older patients, dementia may affect younger individuals as well. Early-onset dementia symptoms develop before the age of 65 [1]. Dementia is caused by damage to or loss of neurons and their connections in the brain. There are multiple etiologies of dementia, but the most common cause is Alzheimer’s disease. A report estimated that around 6.7 million Americans aged 65 and older are living with this disorder [2]. In addition to their profound memory loss, dementia patients develop mood symptoms, including depression, anxiety, hypomania, and psychotic symptoms [3,4]. Management of these issues is important to maintain quality of life, which includes independence and social relationships, making early identification and treatment essential.

A common but largely unstudied mood symptom in dementia patients is pseudobulbar affect (PBA). Dementia patients present with significant cognitive impairment, which is associated with problems in their functioning. However, dementia patients who present with PBA may be misdiagnosed with known psychiatric mood symptoms. Poeck defined four criteria for PBA: (1) the presence of an emotional response inappropriate to the situation; (2) incongruence of emotions and affective response; (3) inability to control the duration and severity of the episode; and (4) emotional expression that does not lead to a feeling of relief for the patient [5]. PBA presents with uncontrolled crying spells or laughter that may or may not be consistent with the social context or connected to the patient’s current emotional state. Though an estimated 1.8 to 7.1 million Americans have experienced PBA in their lifetime, only about 41% of patients who report these symptoms to a physician receive a diagnosis or treatment [6]. This article focuses on the presentation of PBA in patients diagnosed with dementia, but PBA has also been encountered in numerous neurological diseases, including amyotrophic lateral sclerosis, multiple sclerosis, traumatic brain injuries, strokes, brain tumors, Parkinson’s disease, Alzheimer’s disease, and other forms of dementia [7].

To establish a baseline understanding of this issue, we gathered a cohort of patients across multiple health care organizations diagnosed with all-cause dementia who presented with PBA using the TriNetX database. Past studies have documented incidences of PBA in small subsets of patients across several neurocognitive disorders [8,9], but none have specifically examined PBA in dementia patients or utilized a cohort as large as is available through TriNetX. By establishing descriptive statistics for this population, we aim to build a foundation for better identification and treatment of PBA in dementia patients.

## Materials and methods

Given the use of de-identified patient records and the absence of any collection, use, or transmission of individually identifiable data in this retrospective cohort study, it was deemed exempt from institutional review board approval and informed consent, as per the Health Insurance Portability and Accountability Act (HIPAA). Furthermore, we strictly adhered to the reporting guidelines outlined in the Strengthening the Reporting of Observational Studies in Epidemiology (STROBE) framework throughout all phases of our investigation [10].

Eligible patients were identified through TriNetX, a global health research network that provides researchers access to extensive de-identified patient information extracted from the electronic health records (EHRs) of over 250 million patients worldwide. These records are sourced from more than 220 healthcare organizations (HCOs), categorized into subnetworks based on region, capabilities, and data sources. Participating HCOs, primarily academic medical centers, encompass a range of healthcare encounters, including emergency department visits, outpatient care, and inpatient services. Our study focused on a network of 61 HCOs, encompassing over 105 million patients exclusively from the United States.

We included patients aged 19 to 90 years who were part of the TriNetX research network described above, spanning from 2010 to 2023. The age range was defined as ±17 standard deviations (SD) from the peak age of 65, ensuring the inclusion of 100% of ages at which dementia was present.

**Figure 1:**
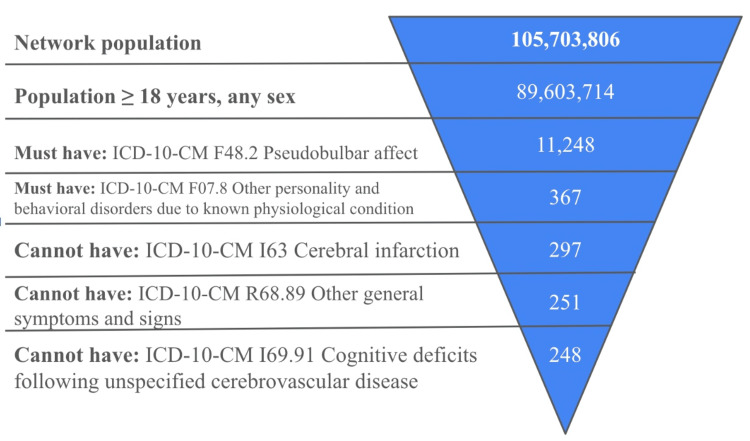
Study flow diagram. Exclusion criteria that did not affect the sample included SNOMED 170600009 Stroke monitoring, ICD-10-CM G46.3 Brain stem stroke syndrome, ICD-10-CM G46.4 Cerebellar stroke syndrome, ICD-10-CM I63.50 Cerebral infarction due to unspecified occlusion or stenosis of unspecified cerebral artery, LOINC 20562-5 Left ventricular stroke volume, and LOINC 20328-1 Left ventricular stroke volume by US second left ventricular outflow tract area calculated.

From this patient set, we formed an exposed cohort of patients with dementia and PBA. Exclusion criteria for the cohorts included a prior diagnosis of stroke before the index event (PBA) or the occurrence of a structural intracranial injury before or after the index event. Inclusion and exclusion criteria were identified using the International Classification of Diseases, 10th Revision (ICD-10) codes (Figure 1). After selecting the cohorts, we computed incidence and prevalence rates using the built-in TriNetX algorithm. The date of the index event (dementia) was considered the date of entry for patients in the cohort. Patients were then followed from their index event until they experienced PBA. Follow-up ranged from five years to a maximum of 15 years.

## Results

**Figure 2:**
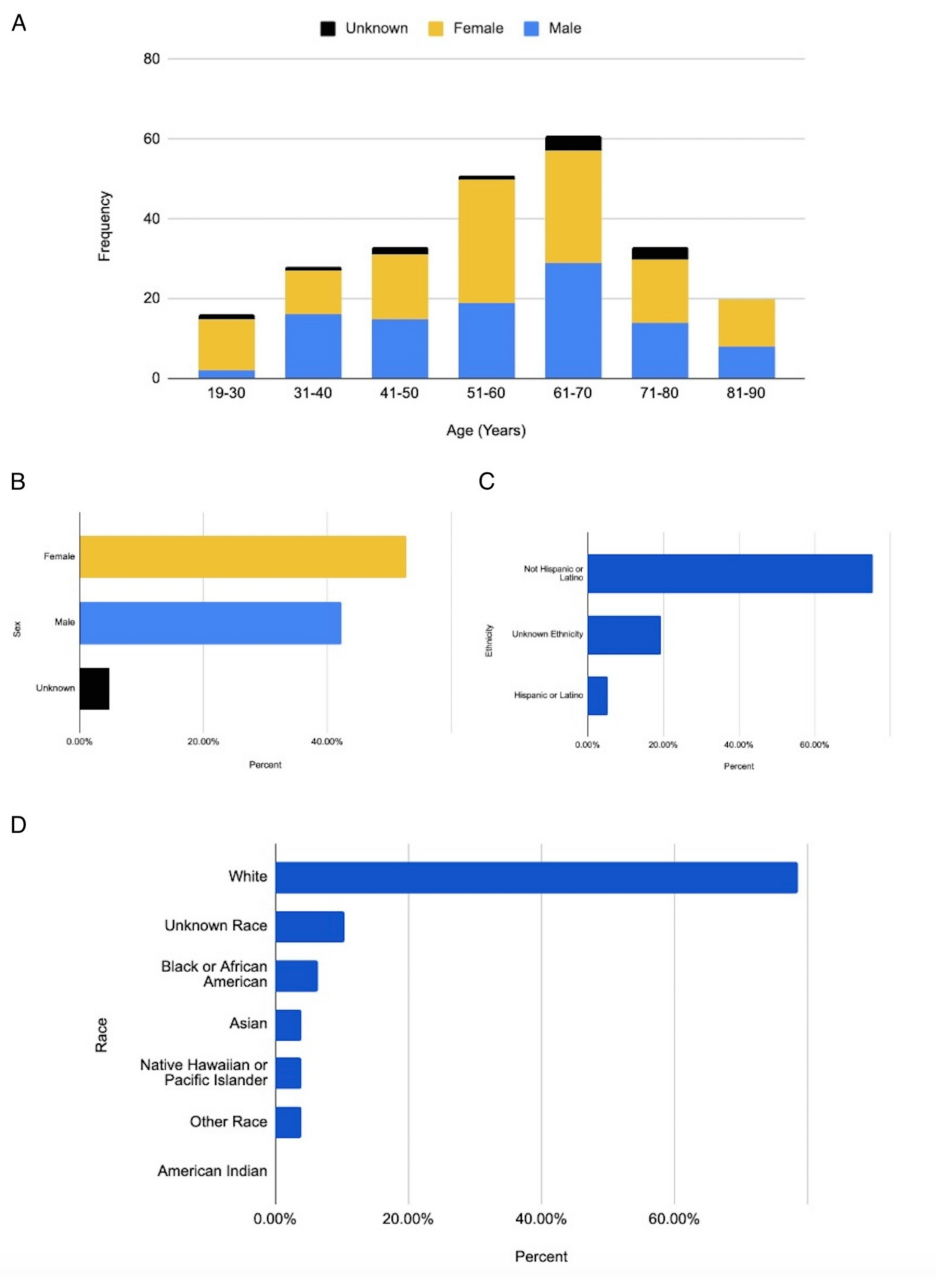
Demographic information. A. Age and gender distribution. B. Sex distribution. C. Ethnicity distribution. D. Race distribution.

The US collaborative network TriNetX includes 105,703,806 patients across 61 healthcare organizations (HCOs). We identified 248 patients with both pseudobulbar affect (PBA) and dementia, applying exclusion criteria that ruled out previous strokes, cerebrovascular injuries, or cerebral infarctions (Figure 1). The time window for the data spans from 2010 to 2023. The demographics of the sample showed a minimum age of 19 and a maximum age of 90, with a mean age of 58 and a standard deviation of 17.53 (Figure 2a). Among the patients, 53% (n=131) were female, 42% (n=104) were male, 4% (n=10) had unknown gender, and 1% (n=3) did not report gender (Figure 2b). Regarding ethnicity, 5% of the patients (n=12) were Hispanic or Latino (Figure 2c). In terms of race, 78% (n=193) were White, 10% (n=25) were of unknown race, 10% (n=25) were Black, and 4% (n=10) were Asian, Native Hawaiian, or other races (Figure 2d).

Patients in this cohort had several common medical diagnoses (Table 1). Seventy-three percent of the cohort (n=182) had postconcussional syndrome, 18% (n=44) had unspecified dementia, 49% (n=121) had a disorder of cognitive function, and 42% (n=105) had some form of amnesia (characterized only by memory loss and not the broader cognitive deficits associated with dementia). Among the common causes of dementia, Alzheimer’s disease was diagnosed in 4% of the cohort (n=10), Parkinson’s disease in 9% (n=22), and multiple sclerosis in 7% (n=17). Many patients also had comorbid psychiatric disorders (Table 2).

**Table 1:** Medical diagnoses. Most common medical diagnoses exhibited in the cohort of comorbid dementia and pseudobulbar affect patients as reported by TriNetX.

Diagnosis	Frequency	Percentage
Postconcussional syndrome	182	73%
Unspecified dementia	44	18%
Other disorders of cognitive function	121	49%
Other amnesia	105	42%
Alzheimer's disease	10	4%
Parkinson's disease	22	9%
Multiple sclerosis	17	7%

**Table 2:** Psychiatric diagnoses. Most common psychiatric diagnoses exhibited by the cohort with comorbid dementia and pseudobulbar affect patients as reported by TriNetX.

Diagnosis	Frequency	Percentage
Reactions to severe stress and adjustment disorders	75	30%
Phobic anxiety disorder	16	6%
Obsessive compulsive disorders	15	6%
Dissociative and conversion disorders	12	5%
Somatoform disorders	10	4%
Other anxiety disorders	137	55%
Mood disorders	168	68%
Major depressive disorder	57	23%
Bipolar disorder	26	10%

Most commonly, 68% (n=168) of the cohort had a type of mood disorder, including major depressive disorder and bipolar disorder. Additionally, 30% (n=75) were diagnosed with reactions to severe stress or adjustment disorders, such as PTSD, and 55% (n=137) were diagnosed with other anxiety disorders. There were also a number of general signs and symptoms observed in this cohort (Table 3). Malaise and fatigue were reported in 47% of patients (n=117), and headache was observed in 42% (n=104). Other symptoms included syncope (21%, n=53), unspecified pain (21%, n=51), convulsions (21%, n=51), and hallucinations (5%, n=12).

**Table 3:** Signs and symptoms. Most common signs and symptoms exhibited by the cohort of comorbid dementia and pseudobulbar affect patients as reported by TriNetX.

Diagnosis	Frequency	Percentage
Malaise, fatigue	117	47%
Headache	104	42%
Dizziness, giddiness	91	37%
Syncope, collapse	53	21%
Unspecified pain	51	21%
Convulsions	51	21%
Restlessness, agitation	27	11%
Suicidal ideation	26	10%
Emotional lability	22	9%
Irritability, anger	16	6%
Hallucinations	12	5%
Low self-esteem	10	4%
Demoralization, apathy	10	4%
Violent behavior	10	4%

Considering the number of comorbidities in this population, these patients were also administered several medications (Table 4). A significant proportion were on pain management medications, including analgesics (81%, n=202) and anesthetics (58%, n=144). Many were also prescribed psychiatric medications, most commonly antidepressants (79%, n=196). Additionally, 85% of these patients (n=212) were on cardiovascular medications, and 84% (n=208) were on respiratory tract medications.

**Table 4:** Medication types. Most common medications taken by the cohort of comorbid dementia and pseudobulbar affect patients as reported by TriNetX.

Medication Type	Frequency	Percentage
Analgesics	202	81%
Antidepressants	196	79%
Sedatives, hypnotics	170	69%
Anticonvulsants	155	63%
Anesthetics	144	58%
Antipsychotics	92	37%
Cardiovascular medications	212	85%
Respiratory tract medications	208	84%

## Discussion

We looked at the characteristics of patients with comorbid PBA and dementia using the TriNetX database. The pathophysiology of PBA is unknown, but it likely involves disruptions in brain structures and neurotransmitter pathways that regulate emotional expression, including dopamine, serotonin, norepinephrine, and glutamate. Disorders that disrupt the circuits projecting to the cerebellum and brainstem may result in disinhibition of normally controlled emotions, making them involuntary [3,8,11,12]. Neurodegenerative conditions such as dementia, especially in their late stages, could disrupt these circuits.

In the context of dementia, PBA can be particularly challenging. The emotional outbursts associated with PBA can be mistaken for behavioral symptoms of dementia, leading to potential misdiagnosis or underdiagnosis. The majority of our cohort were diagnosed with some type of mood disorder in addition to dementia, and instances of PBA could easily be attributed to these conditions. PBA can also interfere with the proper diagnosis of dementia, especially the evaluation of agnosia, aphasia, and apraxia [13]. This can complicate the clinical management of dementia patients and add to their distress and that of their caregivers. One case report describes a female patient with dementia who presented with uncontrollable crying with no apparent trigger [14]. The episodes were initially attributed to depression, but pharmacological treatment for depression was ineffective. Before a diagnosis of PBA was made, these symptoms caused great emotional distress for the patient and her family members alike. Further, in a study of PBA in patients with traumatic brain injury (TBI), Tetano et al. noted that patients with PBA experienced a higher prevalence of anxiety symptoms and poor social functioning compared to patients without PBA [15]. These results are consistent with our finding of overlap between PBA and anxiety disorders. PBA can also interfere with the proper clinical evaluation and management of other disorders. A report of four cases of PBA found that the symptoms made the evaluation of swallowing, proper motor function, and verbal communication much more difficult in a rehabilitation facility [16]. These observations illustrate the importance of proper diagnosis and research of PBA. A better understanding of this symptomatology will facilitate better evaluation and treatment, improving outcomes for these patients.

Management of PBA in dementia involves a combination of pharmacological and non-pharmacological approaches. A systematic review of pharmacological studies of PBA patients with TBI found dextromethorphan/quinidine (DM/Q), now an FDA-approved treatment for PBA, to be consistently effective in reducing PBA symptoms, though studies were limited by high dropout rates and lack of a control group [17]. Antidepressants like citalopram and sertraline may also be used. Non-pharmacological strategies include patient and caregiver education, cognitive-behavioral techniques, and supportive therapies. A report found that physician awareness of PBA was lacking and that the burden of education often fell to patients [18]. These results highlight the need for educational tools for both physicians and patients, especially since the report found education on symptom management significantly improved PBA and associated health outcomes. It is also important to note that the majority of investigations into the effective treatment and management of PBA focus on patients with TBI and/or stroke rather than neurodegenerative conditions such as dementia.

There were several limitations to the present study. As we provided only a descriptive analysis of this cohort, we did not establish any statistically significant relationships between the variables described. Further, though we focused on the subset of dementia patients exhibiting PBA, we recognize that dementia itself is a very broad group. We did not control for the cause of dementia nor differentiate between its etiologies, except under the report of medical diagnoses within the cohort. Another limitation is the lack of control for confounding factors that could lead to PBA, independent of dementia. Finally, as there is no gold standard for PBA diagnosis or recognition, a number of patients in the TriNetX database may have unreported PBA. Even within the broad population of dementia patients, our TriNetX query returned a relatively small sample for those with comorbid dementia and PBA.

PBA can significantly impact the quality of life of dementia patients, affecting their social interactions and overall well-being. Therefore, an accurate diagnosis and effective management of PBA are crucial in the comprehensive care of patients with dementia.

## Conclusions

PBA is a mood symptom seen in patients with dementia, yet few have examined the relationship between these two symptoms or the patient population experiencing them. In the present study, we examined a cohort of these patients using the TriNetX platform. We describe the distribution of medical and psychiatric illnesses, medications, and unrelated symptoms exhibited by the cohort. These results show that clinical recognition and diagnostic criteria for PBA must be established, validated, and implemented to ensure a correct and sound diagnosis of PBA, which will indirectly lead to better patient care.
